# Retraction: Cajanonic acid A regulates the ratio of Th17/Treg via inhibition of expression of IL-6 and TGF-β in insulin-resistant HepG2 cells

**DOI:** 10.1042/BSR-2018-1716_RET

**Published:** 2022-01-20

**Keywords:** Cajanonic acid A, IL-6, TGF-β, Th17/Treg

This Retraction follows an Expression of Concern relating to this article previously published by Portland Press.

This article is being retracted from *Bioscience Reports* at the request of the Editor-in-Chief and the Editorial following receipt of a notification from a reader, alerting the Editorial Board to multiple duplicate data points in the images from Figure 3A. Upon further examination by the Editorial Office, additional clusters of duplicated data points were also noted in in Figure 3F and 4C.

The authors have been contacted in regards to the retraction, and were unable to provide the Editorial Office with the original data which they stated was obtained using equipment from another laboratory, which can no longer be accessed/retrieved due to equipment issues. Given the extent of the issues raised, the Editorial Board stand by the decision to retract the article. The authors agree to the Retraction.

